# Mamushi bites in a kidney transplant recipient

**DOI:** 10.1002/iju5.12460

**Published:** 2022-05-01

**Authors:** Tadasuke Ando, Syunsuke Nakashima, Satoki Abe, Dai Watanabe, Kazunori Iwasaki, Mayuka Shinohara, Tomoki Kai, Shinro Hata, Tadamasa Shibuya, Toshitaka Shin

**Affiliations:** ^1^ Department of Urology, Faculty of Medicine Oita University Yufu Oita Japan

**Keywords:** acute renal failure, immunosuppressive drugs, kidney transplant recipients, mamushi bites, rejection

## Abstract

**Introduction:**

Mamushi bites are the most common venomous snakebites in Japan. The clinical course of a common mamushi bite is known, and its alarming complication and cause of death are acute renal failure due to the venom. However, reports of mamushi bites in kidney transplant recipients are lacking, and the clinical course is unknown.

**Case presentation:**

A 66‐year‐old man who was bitten by a mamushi 3 years after kidney transplantation. Similar to the course of a typical mamushi bite, his severity gradually worsened to its peak 3 days after the bite, after which he turned lightly. A sufficient amount of infusion and continued immunosuppressive drugs were used to avoid acute renal failure.

**Conclusion:**

Even if the mamushi bite occurs in a kidney transplant recipient, the course and management may be the same as usual by continuing the immunosuppressive drugs and a sufficient amount of infusion.

**Abbreviations & Acronyms**
ALT: alanine aminotransferase
AST: aspartate aminotransferase
CPK: creatine phosphokinase
Cr: creatinine
KTR: kidney transplant recipient
LDH: lactate dehydrogenase
MB: mamushi bite
SpO_2_
: oxygen saturation of peripheral artery
TT: tetanus toxoid

**Keynote message**

Mamushi bite occurs in a kidney transplant recipient, the course and management may be the same as usual by continuing the immunosuppressive drugs and a sufficient amount of infusion.

## Introduction

Mamushi is a common venomous snake found in Japan and MB is the most common venomous snakebite in Japan, with 1000–3000 cases per year.^1^, ^2^, ^3^, ^4^, ^5^, ^6^, ^7^, ^8^ The clinical course of a MB gradually progresses; the severity peaks on 3–4 days and then relieves within a few days to 2 weeks, except for some severe cases.^1^, ^2^, ^3^, ^4^, ^5^, ^6^, ^7^, ^8^ Additionally, a high CPK level correlates with MB severity.^1^, ^2^, ^3^, ^4^, ^5^

The mamushi venom includes the following different enzymes: arginine ester dehydrogenase (increased vascular permeability), endopeptidase or bleeding factor (HR1 or HR2) (rhabdomyolysis), an alpha or a beta‐toxin (neurotoxins), hemorrhage 1 and 2, L‐amino acid oxidase 1 and 2 (platelet aggregation), and phospholipase A2 (hemolytic toxins).^5^, ^6^, ^7^, ^8^ Therefore, 3.3% of patients with MB develop acute renal failure due to hypovolemic shock, rhabdomyolysis, and direct renal injury of mamushi venom.^5^, ^6^, ^7^, ^8^ The mortality rate of MB is approximately 0.2–1.0%, and the main cause of death is acute renal failure.^1^, ^2^, ^3^, ^4^, ^5^, ^6^, ^7^, ^8^ Therefore, the main target is to prevent acute renal failure, and appropriate treatment according to severity is important. However, reports of MB in KTRs are lacking, and what the clinical course would be remains unclear. We report on the clinical experience of a KTR who had an MB.

## Case presentation

A 66‐year‐old man, stable 3 years after kidney transplantation in our hospital, felt a tingling pain in his right index finger while weeding a field in the evening of late August and confirmed a mamushi nearby. He was admitted to our hospital immediately. Characteristics, such as two parallel fang marks with dark purple bleeding foci under the skin, were found on his right index finger. The redness and swelling were just localized to the right hand. Upon arrival at the hospital, his consciousness was clear, with a blood pressure of 140/70 mmHg, pulse of 70 beats per minute, body temperature of 36.5°C, and SpO_2_ of 99% under room air. He did not report double vision.

Judging from these situations comprehensively, we diagnosed him as MB grade I. The biochemical analysis results of the blood performed on arrival were as follows: white blood cells 9800/μL, hemoglobin 10.8 g/dL, platelets 21.0 × 104/μL, total protein 6.0 g/dL, glucose 121 mg/dL, AST 12 IU/L, ALT 6 IU/L, blood urea nitrogen 21.9 mg/dL, Cr 2.1 mg/dL, sodium 139 mEq/L, potassium 4.8 mEq/L, chloride 101 mEq/L, CPK 34 IU/L, LDH 245 IU/L.

The administration of lactated Ringer's solution infusion (3000 mL/day), cepharanthin, antibiotics, and TT was initiated according to the dosage stated in the package insert. However, due to his strong allergic reaction to the manufactured mamushi antivenom, it could not be administered. Tacrolimus, mycophenolate mofetil, and methylprednisolone, which had been used as immunosuppressive drugs after kidney transplantation, were continued at their prescribed doses.

**Fig. 1:**
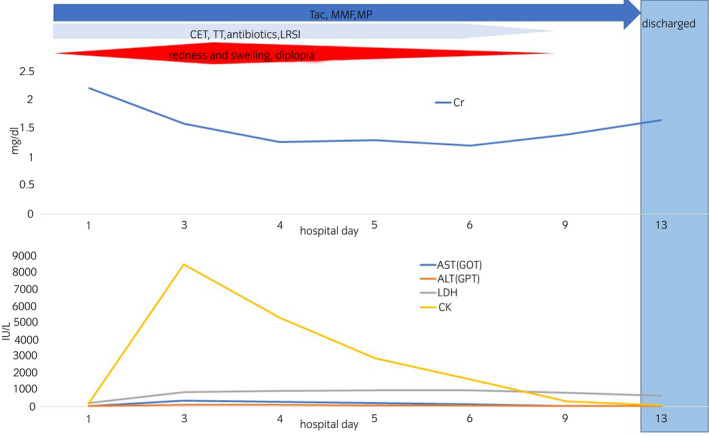
Clinical course of a KTR bitten by mamushi biochemical results and systematic symptom gradually deteriorated until the third day, and then turned to a lightly as previously reported. [Colour figure can be viewed at wileyonlinelibrary.com]

After admission, the area of redness and swelling continued to expand, and on the third day, these areas extended to the chest and diplopia appeared. We determined that the MB grade worsened to V. Similarly, laboratory data suggested rhabdomyolysis with a high CPK level (Fig. 1). The severity grade worsened, but the same treatments were continued. Fortunately, however, urine output and renal function were adequately maintained.

After the fourth hospital day, MB grade and biochemical results of the blood gradually improved. He was discharged on hospital day 14 without any deterioration of renal function or infection or any signs of disseminated intravascular coagulation.

## Discussion

This patient course provided two important clinical suggestions.

First, this case followed the same clinical course as previously reported general MB patients: a severe peak of MB grade, rhabdomyolysis with a high CPK level, widespread edema extending to dryness, and diplopia 3 days after MB.^1^, ^2^, ^3^, ^4^, ^5^, ^6^, ^7^, ^8^ These were gradually relieved over the next 10 days.^1^, ^2^, ^3^, ^4^, ^5^, ^6^, ^7^, ^8^ Therefore, this case suggests that it is possible to treat MBs in KTRs according to the severity reported before and predict the clinical course.^1^, ^2^, ^3^, ^4^, ^5^, ^6^, ^7^, ^8^ Grade classification for MBs^1^, ^2^, ^3^, ^4^, ^5^, ^6^, ^7^, ^8^ is clinically used to determine the severity of injuries as follows: Grade I, redness and swelling localized at the bitten area; Grade II, redness and swelling limited to the wrists and ankles; Grade III, redness and swelling limited to the elbow or knee joint; Grade IV, redness and swelling of the whole extremity; and Grade V, redness and swelling in parts beyond the extremity or exhibiting systemic symptoms including diploma. The manufactured mamushi antivenom treatment is definitive and recommended for Grade III or higher defined as severe.^1^, ^2^, ^3^, ^4^, ^5^, ^6^, ^7^, ^8^

Second, this case did not need to reduce or discontinue immunosuppressive drugs.

Mamushi venom is a variety of enzymes that act directly on vascular permeability, muscle, platelets, and nerves, and it is speculated that immunosuppression by immunosuppressive drugs does not enhance the effect of mamushi venom. Since in general, rejection is the main cause of renal transplant loss, KTRs should not discontinue immunosuppressive drugs unless they have a malignant tumor or an uncontrollable infectious disease.^9^ Additionally, KTRs are in a chronic kidney disease state and are prone to renal damage due to dehydration.^9^ From the viewpoint of preventing acute renal failure, immunosuppressive drugs should be continued even in severe MB case, and a sufficient amount of infusion is required to prevent rejection and dehydration. So far, no special infection has been found in patients who have MBs reported before, and administering TT in all cases is unnecessary.^8^ In our case, despite the severe case in which the manufactured mamushi antivenom could not be administered, the disease improved without renal failure as complication and rejection with continued immunosuppressive drug and sufficient infusion. The clinical course was almost the same as a general MBs.

## Conclusion

KTRs with MB followed a similar clinical course as general MB patients and did not require dose reduction or discontinuation of immunosuppressive drugs during treatment. In the future, it is possible that a new KTR with MB will occur. Our case should be considered a valuable report for treating KTRs with MB. Thus, it is necessary to accumulate case reports, such as our cases.

## Author contributions

Tadasuke Ando: Conceptualization; data curation; formal analysis; investigation; methodology; project administration; writing – original draft. Syunsuke Nakashima: Data curation. Satoki Abe: Data curation. Dai Watanabe: Data curation. Kazunori Iwasaki: Data curation. Mayuka Shinohara: Data curation. Tomoki Kai: Data curation. Shinro Hata: Data curation. Tadamasa Shibuya: Data curation. Toshitaka Shin: Supervision.

## Conflict of interest

The authors declare no conflict of interest.

## Approval of the research protocol by an Institutional Reviewer Board

N/A.

## Informed consent

N/A.

## Registry and the Registration No. of the study/trial

N/A.
